# Low Fluence Ultraviolet-B Promotes Ultraviolet Resistance 8-Modulated Flowering in *Arabidopsis*

**DOI:** 10.3389/fpls.2022.840720

**Published:** 2022-03-31

**Authors:** Anna Zioutopoulou, Eirini Patitaki, Liz O’Donnell, Eirini Kaiserli

**Affiliations:** College of Medical, Veterinary and Life Sciences, Institute of Molecular, Cell and Systems Biology, University of Glasgow, Glasgow, United Kingdom

**Keywords:** light, UV-B, *Arabidopsis*, flowering, photoperiod

## Abstract

Ultraviolet-B (UV-B) irradiation (280–320 nm) is an integral part of sunlight and a pivotal environmental cue that triggers various plant responses, from photoprotection to photomorphogenesis and metabolic processes. UV-B is perceived by ULTRAVIOLET RESISTANCE 8 (UVR8), which orchestrates UV-B signal transduction and transcriptional control of UV-B-responsive genes. However, there is limited information on the molecular mechanism underlying the UV-B- and UVR8-dependent regulation of flowering time in plants. Here, we investigate the role of UV-B and UVR8 in photoperiodic flowering in *Arabidopsis thaliana*. Our findings suggest that UV-B controls photoperiodic flowering in an ecotype-specific manner and that UVR8 acts as a negative regulator of UV-B-induced flowering. Overall, our research shows that UV-B modulates flowering initiation through the action of UVR8 at the transcriptional level.

## Introduction

Plants are sessile organisms that have evolved to adapt to environmental variation in order to ensure survival. Light, in particular, is an external stimulus that affects every aspect of a plant’s life, from seed germination to the transition to the reproductive state by floral initiation (Sullivan and Deng, 2003). Plants are able to perceive the intensity, quality, duration, and even direction of light in order to phenotypically adjust according to their ever-changing environment (Montgomery and Lagarias, 2002). However, since plants have not evolved eyesight like organisms that belong to the animal kingdom, they employ proteins called photoreceptors which perceive light cues, allowing plants to respond to diurnal and seasonal light shifts (Briggs and Olney, 2001). This sophisticated mechanism grants the ability for plants to timely coordinate crucial developmental processes such as flowering initiation (Fornara et al., 2010). *Arabidopsis thaliana* is a facultative long-day plant and its flowering time is mediated through the function of two master orchestrators: FLOWERING LOCUS T (FT) and CONSTANS (CO) that are regulated by light, circadian rhythms, temperature, and hormones (Fornara et al., 2010). CO induces the expression of *FT* in a long-day-dependent fashion, through direct association with its promoter (Fornara et al., 2010). *CO* gene expression and protein stability is tightly regulated by light and circadian clock components (Imaizumi et al., 2005; Sawa et al., 2007; Song et al., 2012). For instance, FLAVIN-BINDING, KELCH REPEAT, F-BOX 1 (FKF1) and GIGANTEA (GI) facilitate the degradation of CYCLING DOF FACTOR (CDF) proteins which suppress the expression of *CO* and *FT* (Sawa et al., 2007; Song et al., 2012). Furthermore, GI stabilizes FKF1 and ZEITLUPE (ZTL), which act in synergy with their homolog LOV KELCH PROTEIN 2 (LKP2) to degrade the DOF transcription factor CDF2 (Kim et al., 2007; Fornara et al., 2009). An additional circadian clock component that controls CO abundance is EARLY FLOWERING 3 (ELF3) (Hicks et al., 2001; Nusinow et al., 2011). In particular, ELF3 forms a complex with CONSTITUTIVE PHOTOMORPHOGENIC 1 (COP1) and GI which leads to the inactivation of GI and ultimately the repression of *CO* expression (Zhao et al., 2021).

Ultraviolet-B irradiation is an integral part of sunlight that reaches the earth and ranges from 280 to 315 nm. Although UV-B can be harmful to most living organisms, it simultaneously triggers various photomorphogenic responses in plants depending on the intensity (Jenkins, 2014). Plants perceive UV-B light through the function of UVR8, the only photoreceptor identified so far absorbing and mediating responses to UV-B light (Brown et al., 2005; Favory et al., 2009; Rizzini et al., 2011; Jenkins, 2014). Upon UV-B exposure, dimeric UVR8 undergoes structural alterations that lead to the photo-induced dissociation of the dimer into a monomeric active state (Heilmann et al., 2016). UVR8 activation is negatively regulated by REPRESSOR OF UV-B PHOTOMORPHOGENESIS 1 and 2 (RUP1 and RUP2), which facilitate the re-dimerization of the UVR8 monomers (Gruber et al., 2010; Heijde and Ulm, 2012). Upon monomerization, UVR8 translocates from the cytosol to the nucleus, where it interacts with COP1 an E3 ubiquitin ligase, to mediate photomorphogenic and photoprotection responses through the transcriptional activation of UV-B-responsive genes (Kaiserli and Jenkins, 2007; Cloix and Jenkins, 2008; Heijde and Ulm, 2012). In addition, the physical interaction between UVR8 and COP1 leads to the stabilization of bZIP transcription factor ELONGATED HYPOCOTYL 5 (HY5), granting the activation of HY5-induced genes involved in UV-B-associated photomorphogenic responses (Ulm et al., 2004; Huang et al., 2012).

UVR8 has been reported to antagonize shade avoidance and thermomorphogenic responses through the deactivation of PHYTOCHROME INTERACTING FACTOR 4 and 5 (PIF4 and PIF5) (Hayes et al., 2014, 2017). A recent study demonstrated that even in the absence of shade conditions the UVR8-mediated degradation of PIF4 and PIF5 is an essential step in UV-B signal transduction and UVR8-dependent hypocotyl growth inhibition (Tavridou et al., 2020).

Apart from photomorphogenic responses, low intensity UV-B also mediates circadian clock entrainment through the synergistic function of UVR8 and COP1 (Feher et al., 2011). However, circadian clock components can in turn attenuate UV-B responsiveness by repressing *UVR8*, *COP1*, and UV-B-induced genes to limit energy expenses (Feher et al., 2011). Indicatively, studies on arrhythmic *elf3* mutants showed elevated levels of UV-B-associated gene activation, however, this response did not promote UV-B stress tolerance (Feher et al., 2011).

Studies on the role of UV-B and/or UVR8 in regulating flowering time in plants are limited. *Landsberg erecta* (*Ler*) wild type plants subjected to UV-B irradiation demonstrated a delayed flowering phenotype, whilst the opposite was observed in *uvr8* mutants which flower earlier than their wild type counterparts (Hayes et al., 2014). In addition, the delay in flowering time in the *Arabidopsis Columbia-0* (*Col-0*) ecotype under long day (LD) and short day (SD) photoperiodic conditions has also been attributed to the UVR8-signaling trajectory. Flowering time analysis demonstrated that *uvr8* did not show a significant change in flowering time compared to a late flowering phenotype observed in *Col-0* in response to WL (white light) supplemented with short-time interval high fluence rate UV-B in LD (Dotto et al., 2018). Interestingly, the UVR8-repressor, RUP2, was shown to inhibit CO from binding to the *FT* promoter leading to a significant delay in flowering time in plants under a SD photoperiod supplemented with UV-B (Arongaus et al., 2018).

Although a lot of effort has been invested in elucidating how UVR8 and UV-B regulate plant responses, the molecular mechanism through which the aforementioned factors regulate flowering time under LDs remains largely uncharted. Our results indicate that constant low levels of UV-B (0.5 μmol m^–2^s^–1^) promote flowering initiation under LD photoperiodic conditions in *Col-0* and *Ler Arabidopsis* ecotypes. Additionally, UV-B induces early flowering of mutants lacking key flowering (*co*), UV-B (*uvr8* and *rup1rup2*) and light signaling (*cop1, pif4*, and *ztl*) components. Furthermore, we show that UVR8 acts as a negative regulator of UV-B-induced early flowering since *uvr8* mutants exhibited early flowering phenotypes under white light supplemented with UV-B. Overall, our data uncover that UV-B can modulate flowering initiation through the action of UVR8 at the transcriptional level.

## Materials and Methods

### Plant Materials and Growth Conditions

The following wild type *Arabidopsis thaliana* ecotypes of *Col-0*, *Ler*, and *Cape Verde Islands* (*Cvi*) were used. Additionally, mutant and transgenic lines were used for flowering and gene expression studies in *Col-0* background [*rup1rup2* (Gruber et al., 2010; Vanhaelewyn et al., 2016), *cop1-4* (McNellis et al., 1994), *constans-10 (co-10)* (Laubinger, 2006), *ztl lkp2 fkf1* (*zlf*) (Baudry et al., 2010), *elf3-1* (Zagotta et al., 1992), *pif4* (Koini et al., 2009), *pif4pif5* (Lucas et al., 2008), and *OX-PIF4-HA* (Kumar et al., 2012)], or *Ler* [*uvr8-1* (Kliebenstein et al., 2002), *uvr8-2* (Brown et al., 2005) and *OX-GFPUVR8*/*uvr8-1* (Kaiserli and Jenkins, 2007)]. The UVR8 over-expressing line will be referenced in the text as OXUVR8. Seeds used for flowering and gene expression experiments were stratified in sterile distilled dH_2_O for 3–4 days at 4°C and were sown on soil (Phytotron growth chambers) under long day (LD) photoperiods (16 h light and 8 h dark) with an illumination intensity of white light (50 μmol m^–2^s^–1^) ± UV-B (0.5 μmol m^–2^s^–1^) for UV-B experiments. The WL in these experiments was provided by LED lights (CEC). UV-B was provided by narrowband fluorescent lights (PHILIPS NARROWBAND TL 40W/0I-PS). The temperature of the chamber plants were grown was 22°C.

### RNA Extraction and Quantitative Gene Expression Analysis

Total RNA was extracted from 12-day-old (until they reached the juvenile phase, before transition to reproductive growth) plants grown under LD photoperiodic conditions at zeitgeber ZT 0.5 (30 min after light onset) and ZT 15 (15 h after light onset). The tissue was rapidly frozen in liquid nitrogen and disrupted using a TissueLyser by Qiagen (1 min with 18.0 m/s frequency). RNA was extracted using the RNeasy plant mini kit (Qiagen) according to the manufacturer’s instructions and the total amount of RNA was quantified using a spectrophotometer nanodrop (Implen). Complementary DNA synthesis was performed on 1 μg of total RNA using the QuantiTect Reverse Transcription Kit by Qiagen according to the manufacturer’s instructions. Real-time quantitative PCR was performed using the StepOnePlus™ Real-Time PCR System (Applied Biosystems, Life Technologies) and the Brilliant III Ultra-Fast SYBR^®^ Green QPCR Master Mix (Agilent Technologies) using the following thermocycler (Step 1: Incubate at 95°C for 2 min, Step 2: Incubate at 95°C for 3 s, Step 3: Incubate at 59.5°C for 30 s, Step 4: Repeat Step 2 and 3 for 50 cycles, Step 5: Incubate at 95°C for 1 min, Step 6: Incubate at 60°C for 30 s, Step 7: Incubate at 95°C for 30 s). Expression of one of two abundantly expresses housekeeping genes was used for normalization. These genes were either *IRON-SULFUR CLUSTER ASSEMBLY PROTEIN 1* (*ISU1*), a well-established house-keeping gene which is involved in iron-sulfur cluster biogenesis (Perrella et al., 2018) or *ISOPENTENYL DIPHOSPHATE ISOMERASE 2* (*IPP2*), another well-established house-keeping gene used as a control for clock and light signaling gene expression studies as its expression is not regulated by diurnal rhythms (Farre et al., 2005; Zhu et al., 2016; Hwang et al., 2017). The amplification efficiency of each sample was calculated by StepOne™ Software v2.2 (Life Technologies) by using the slope of the regression line in the standard curve. The normalization of the data was achieved by geometric averaging of *ISU1* or *IPP2* as internal reference genes. A complete list of primers used for qRT-PCR are listed in Supplementary Table 1.

### Flowering Time Measurements

Flowering time was monitored by either (a) counting the total number of rosette leaves of each plant on the day it bolted (appearance of the first flower bud with a stem of 2 cm) or (b) by calculating the number of days after germination at the time of bolting.

### Statistical Analysis

The statistical analysis of the results acquired in the current research was performed using Student’s *t*-test provided by Excel. ANOVA analysis was performed using GraphPad Prism 8.4.1 and Tukey’s *Post hoc* test was conducted.

## Results

### Ultraviolet-B Accelerates Photoperiodic Flowering in an Ecotype-Specific Manner

Environmental regulation of flowering time is a complex process that requires signal integration (Fornara et al., 2010). In order to better understand the effect of UV-B irradiation in *Arabidopsis thaliana*, we conducted flowering experiments on different ecotypes, mutants and overexpressing lines of light signaling and flowering components under a LD photoperiod of WL (50 μmol m^–2^s^–1^) or WL supplemented with physiologically relevant UV-B irradiation (0.5 μmol m^–2^s^–1^). The intensity of UV-B irradiation was calculated based on the intensity of UV-B on a sunny day at the University of Glasgow campus (0.5–1 μmol m^–2^s^–1^). Separate measurements were conducted in the months of March, April, May, June, July, and September and a mean of total UV-B intensity at floral level was calculated based on the aforementioned measurements. Three possible UV-B intensities were initially tested (0.3, 0.5, and 1 μmol m^–2^s^–1^) and one of them was chosen (0.5 μmol m^–2^s^–1^) based on its efficiency in mediating UV-B-dependent photomorphogenesis, by monitoring the expression of *HY5*, a marker gene for UV-B mediated photomorphogenic responses (Brown et al., 2005) and was therefore used as an indicator that our UV-B treatment efficiently induced physiological UV-B responses (Supplementary Figures 1A,B). From the intensities we tested 0.3 μmol m^–2^s^–1^ was not able to initiate plant photomorphogenic responses (Supplementary Figure 1C). Moreover, the UV-B intensity selected (0.5 μmol m^–2^s^–1^) did not cause any damaging effects on the plants, in order to avoid stress-induced responses (Supplementary Figure 2). The constant UV-B irradiation of 1 μmol m^–2^s^–1^ was too damaging for plants to survive and could therefore not reach the floral induction stage. Three common *Arabidopsis* ecotypes were tested in our experiments: *Landsberg erecta* (*Ler*), *Columbia-0* (*Col-0*) and *Cape Verde islands* (*Cvi*). In order to assess flowering time two variables were taken into consideration: (a) the number of rosette leaves each plant had on the day the bolt reached approximately 2 cm in height and (b) the number of days after germination when the first bud emerged. From these two parameters the number of rosette leaves was selected as the most reliable assay in order to investigate changes regarding flowering initiation in response to UV-B. This decision was based mainly on two factors. Firstly, flowering experiments in published literature use primarily the number of rosette leaves at the time of bolting to avoid any growth rate defects presented in many mutant genotypes (Wollenberg et al., 2008; Hayes et al., 2014). Furthermore, it is well-established that UV-B inhibits hypocotyl elongation (Gruber et al., 2010) but also delays plant growth altogether which can result in an increase in the number of days that have passed before bolting, which is not directly related to flowering initiation but to growth rate. Nevertheless our conclusions were formed after investigating both of these factors along with data from gene expression analysis.

**FIGURE 1:**
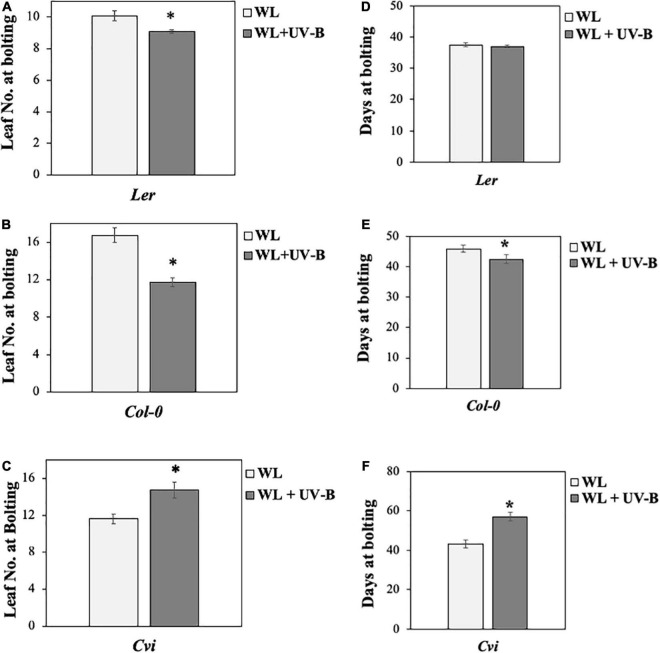
UV-B induces early flowering in *Col-0* and *Ler* but delays it in *Cvi*. **(A–C)** Flowering times (as measured by rosette leaf number) of *Col-0* **(A)**, *Ler* **(B)**, and *Cvi* **(C)** ecotypes grown under a LD photoperiod in WL (50 μmol m^–2^s^–1^) or WL supplemented with UV-B (0.5 μmol m^–2^s^–1^). Data are represented as mean ± SEM (*n* ≥ 15 plants recorded) and an asterisk (*) indicates statistically significant differences (*P* < 0.05) between means. **(D–F)** Flowering times (as measured by the number of days prior to bolting) of *Col-0* **(D)**, *Ler* **(E)**, and *Cvi* **(F)** ecotypes grown under a LD photoperiod in WL (50 μmol m^–2^s^–1^) or WL supplemented with UV-B (0.5 μmol m^–2^s^–1^). Data are represented as mean ± SEM (*n* ≥ 15 plants recorded) and an asterisk (*) indicates statistically significant differences (*P* < 0.05) between means. Data are representative of three biological repeats including all genotypes represented above, a minimum number of 15 plants was assayed per genotype per condition.

**FIGURE 2:**
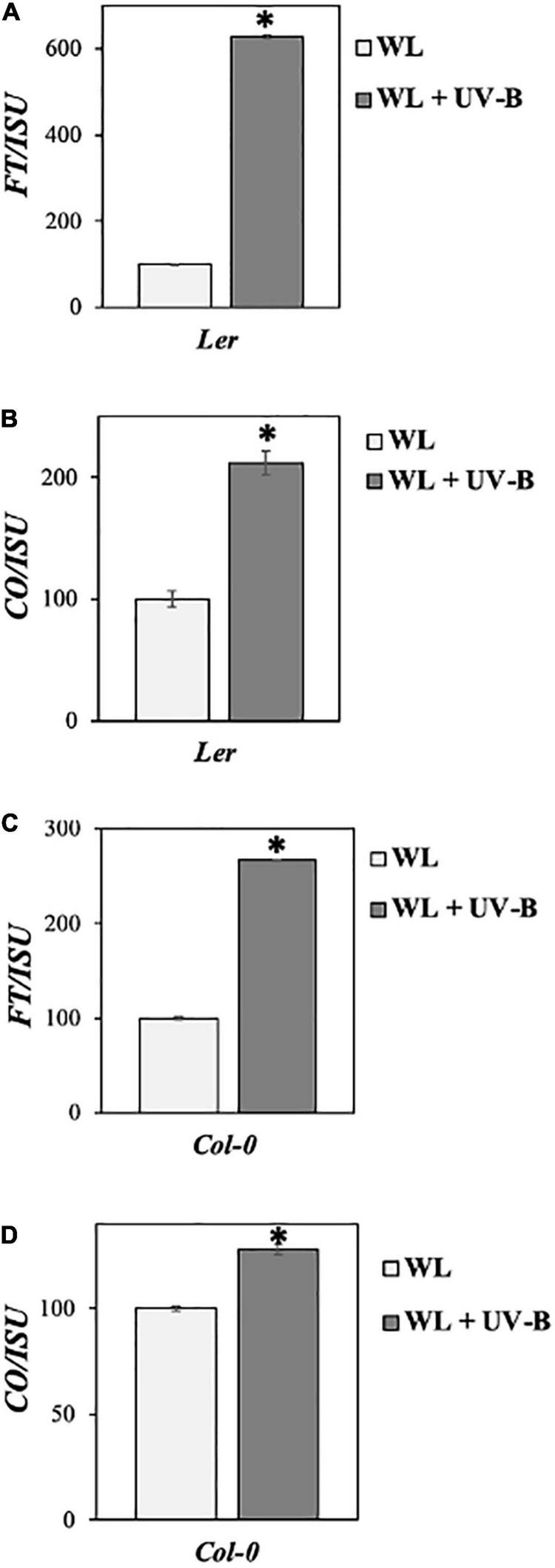
UV-B induces photoperiodic pathway genes *FT* and *CO* in *Col-0* and *Ler* ecotypes. **(A–D)** qRT-PCR analysis of *FT* and *CO* transcript levels in *Ler* **(A,B)** and *Col-0* **(C,D)** normalized with the housekeeping gene *ISU.* Plant tissue was collected at ZT 0.5 12 days after germination. Plants were grown under a LD photoperiod of WL (50 μmol m^–2^s^–1^) or WL supplemented with UV-B (0.5 μmol m^–2^s^–1^). Plants grown under WL were used as reference (100%). Data are represented as mean ± SEM. Data are representative of three biological repeats including all genotypes represented above. For each biological repeat three technical qPCR replicates were conducted. *indicates a statistically significant difference.

Our data showed that flowering time under WL supplemented with UV-B was induced early in *Col-0* wild-type ecotype compared to non-UV-B treated plants, since a statistically significant decrease both in the number of rosette leaves as well as in the number of days before plants bolted was observed under WL supplemented with UV-B versus WL treatments (Figures 1B,E). Wild type *Ler* ecotype also depicted a milder early flowering phenotype, since there was a statistically significant decrease in the number of rosette leaves plants had at the day of bolting but not in the number of days at bolting under UV-B irradiation compared to WL treatment only (Figures 1A,D). Flowering time under UV-B irradiation compared to WL treatment was delayed in the *Cvi* ecotype, more specifically a statistically significant increase was observed in both the number of rosette leaves as well as in the number of days before plants bolted (Figures 1C,F). To further investigate the molecular mechanism underlying the early flowering phenotypes observed in the *Col-0* and *Ler* ecotypes, gene expression analysis was performed monitoring master integrators of the photoperiodic pathway, which is controlled by the day length and the circadian clock. The zeitgeber time point (ZT 0.5) was chosen based on the expression patterns of the monitored genes (Fornara et al., 2010) and at the time point where the maximal UV-B effect was observed. Specifically under LD both *FT* and *CO* expression peaks after dawn (ZT 0.5) and at dusk (ZT 15) (Yanovsky and Kay, 2003). Our results suggest that *FT* and *CO* are significantly upregulated in *Col-0* and *Ler* grown under WL supplemented with UV-B, in comparison to the ones grown solely under WL (Figures 2A–D) indicating that FT and CO are mediating the early induced flowering phenotype observed in *Col-0* and *Ler* under WL supplemented UV-B. On the other hand, *Cvi* plants show a downregulation in *FT* transcript levels (Supplementary Figure 3).

### Ultraviolet Resistance 8 Acts as a Negative Regulator of Flowering

**FIGURE 3:**
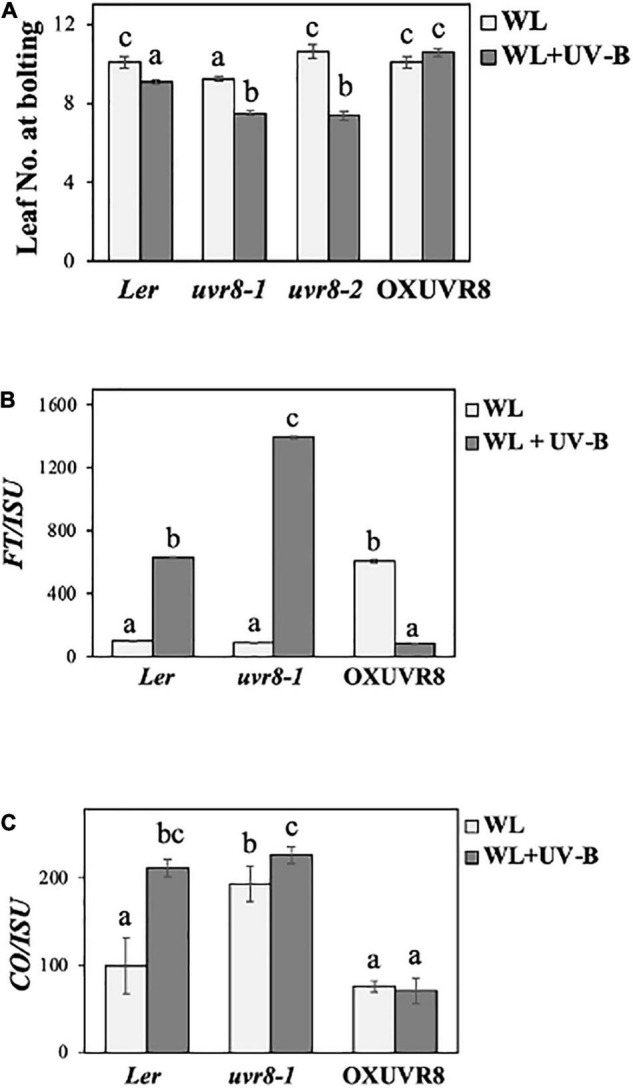
UVR8 acts as a negative regulator of flowering initiation. **(A)** Flowering time of wild-type (*Ler*), *uvr8-1*, *uvr8-2, and* OX-UVR8 plants grown under a LD photoperiod in WL (50 μmol m^–2^s^–1^) or WL supplemented with UV-B (0.5 μmol m^–2^s^–1^). Data are represented as mean ± SEM (*n* ≥ 15). Different letters indicate statistically significant differences (*P* < 0.05) between means. **(B,C)** qRT-PCR analysis of *FT* **(B)** and *CO* **(C)** transcript levels normalized with the housekeeping gene *ISU* in wild-type (*Ler*), *uvr8-1*, *uvr8-2* and OX-UVR8 plants. Plant tissue was collected at ZT 0.5 12 days after germination. Plants were grown under a LD photoperiod of WL (50 μmol m^–2^s^–1^) or WL supplemented with UV-B (0.5 μmol m^–2^s^–1^). Plants grown under WL were used as reference (100%). Data are representative of three biological repeats including all genotypes represented above, a minimum number of 15 plants was assayed per genotype. For qPCR experiments for each biological repeat three technical qPCR replicates were conducted.

Since UVR8 is the only identified UV-B photoreceptor to date (Tilbrook et al., 2013), we were interested in further investigating its role in photoperiodic-controlled flowering initiation, by examining *uvr8* mutant and UVR8 over-expressing lines. We conducted flowering experiments under a LD photoperiod of WL (50 μmol m^–2^s^–1^) or WL supplemented with UV-B (0.5 μmol m^–2^s^–1^), in *Ler* wild-type plants, two different *uvr8* mutant alleles (*uvr8-1* and *uvr8-2*) and a UVR8 over-expressing line. More specifically, to examine the *Ler* ecotype we used a UVR8 over-expressing line in an *uvr8* mutant background [OXUVR8 = *35Spro*GFP-UVR8/uvr8-1 (Kaiserli and Jenkins, 2007)] and two different *uvr8* mutant alleles: *uvr8-1* and *uvr8-2*. *Uvr8-1* mutants have a single recessive mutation leading to a deletion of 15 nucleotides, which results in the absence of UVR8 protein production (null allele) (Kliebenstein et al., 2002). *Uvr8-2* mutants contain a premature stop codon on the 400th amino acid (Brown et al., 2005; Yin et al., 2015), therefore these mutants are still able to produce truncated but non-functional UVR8 protein (Cloix et al., 2012). The data from the flowering experiments depicted that UV-B induces an early flowering phenotype in *uvr8-1* and *uvr8-2*, but does not significantly affect flowering of OXUVR8 (Figure 3A). Additionally, under WL *uvr8-1* flowers earlier than wild type, while *uvr8-2*, OXUVR8 and wild type (*Ler*) flower simultaneously (Figure 3A). When *uvr8-1* and *uvr8-2* mutants were exposed to WL + UV-B, they flowered earlier compared to wild type, while OXUVR8 had a late flowering phenotype (Figure 3A). In order to investigate these responses at the molecular level, we examined once more the transcript levels of genes encoding the key flowering regulators *FT* and *CO* (Fornara et al., 2010) in WT, *uvr8-1* and OXUVR8 backgrounds. Our results indicate that *FT* and *CO* genes are significantly upregulated in *uvr8-1* mutants grown under WL supplemented with UV-B (Figures 3B,C). *FT* transcript levels are also significantly downregulated in the OXUVR8 line in plants grown under WL supplemented with UV-B compared to the ones grown solely under WL, while *CO* levels do not depict significant change (Figures 3B,C). As demonstrated in Figures 3B,C, there is a greater induction of *FT* in *uvr8-1* compared to the wild type and an overall higher level of *FT* transcripts over *CO* in both wild type and mutant plants.

### Ultraviolet-B Affects Flowering in Photoperiodic and Light Signaling Mutant and Over-Expressing Lines

**FIGURE 4:**
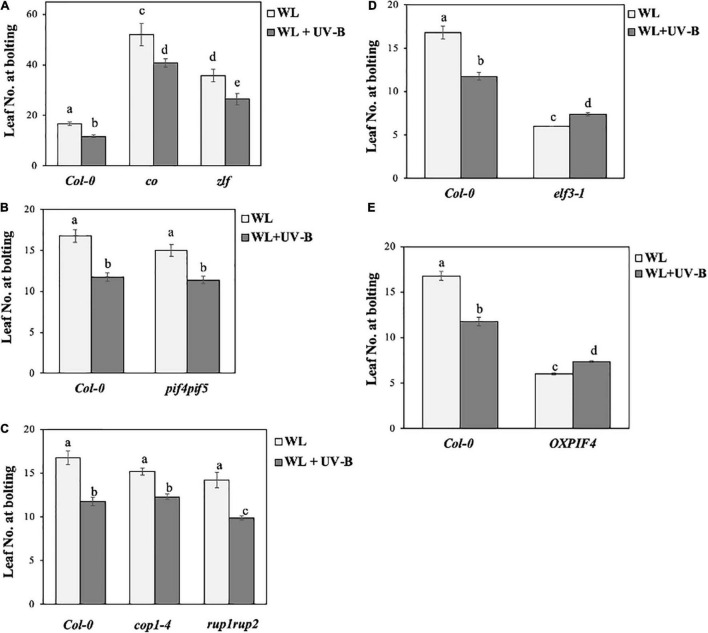
UV-B accelerates flowering of *co*, *zlf*, *pif4pif5*, *cop1-4*, and *rup1rup2* but delays flowering of *elf3-1* and OXPIF4. **(A–C)** Flowering time of wild-type *Col-0*, *co* and *zlf* **(A)**, *pif4pif5* **(B)**, and *cop1-4* and *rup1rup2* **(C)** plants grown under a LD photoperiod in WL (50 μmol m^–2^s^–1^) or WL supplemented with UV-B (0.5 μmol m^–2^s^–1^). Data are represented as mean ± SEM (*n* ≥ 15). Different letters indicate statistically significant differences (*P* < 0.05) between means. **(D,E)** Flowering time of wild-type *Col-0*, *elf3-1* **(D)** and OXPIF4 **(E)** plants grown under a LD photoperiod in WL (50 μmol m^–2^s^–1^) or WL supplemented with UV-B (0.5 μmol m^–2^s^–1^). Data are represented as mean ± SEM (*n* ≥ 15). Different letters indicate statistically significant differences (*P* < 0.05) between means. Data are representative of three biological repeats including all genotypes represented above, a minimum number of 15 plants was assayed per genotype.

To understand how UV-B regulates photoperiodic flowering in Arabidopsis, we examined flowering time initiation of mutant and transgenic lines of key light signaling and/or photoperiodic flowering components. More specifically, *co*, *zlf* and *elf3-1* mutants, lacking photoperiodic flowering components CO (Fornara et al., 2010), ZTL/LKP2/FKF1 (Kim et al., 2007) and ELF3 (Hicks et al., 2001) were examined. *Pif4pif5* mutants lacking the key light signaling proteins PIF4 and PIF5 (Hayes et al., 2014), as well as OXPIF4 over-expressing PIF4 (Kumar et al., 2012) were also examined. Finally, we tested how UV-B dependent flowering in *cop1-4* and *rup1rup2* mutants which lack important UV-B signaling mediating proteins COP1 (Oravecz, 2006; Yin and Ulm, 2017), and RUP1 and RUP2 (Arongaus et al., 2018). The research findings from our flowering experiments showed that UV-B can still induce early flowering in the late flowering *co* and *zlf* mutants, in a similar manner to *Col-0* (Figure 4A). On the contrary, UV-B exposure slightly delayed the early flowering phenotype of *elf3-1* (Figure 4D). Furthermore, UV-B induced an early flowering phenotype in the light signaling *pif4pif5* mutants, which was comparable to the response observed in wild type *Col-0* plants (Figure 4B). Early flowering OX-PIF4 grown under WL and WL supplemented with UV-B, showed a reversion of the early flowering phenotype induced by UV-B that is observed in the wild type *Col-0* (Figure 4E), therefore UV-B is found to also slightly delay flowering initiation in OXPIF4 plants. Finally, differences in flowering time between UV-B signaling *cop1-4* and *rup1rup2* mutants grown under WL and WL supplemented with UV-B, suggest that UV-B induces early photoperiodic flowering in those lines, a similar response to the one observed in wild type *Col-0* plants (Figure 4C).

## Discussion

### The Effect of Ultraviolet-B on Photoperiodic Flowering Is Ecotype-Specific

Understanding the molecular trajectory and components that modulate photoperiodic flowering is key for acquiring the ability to improve plant yield in agricultural production systems. Although UV-B is an integral part of sunlight, the impact of UV-B radiation on photoperiodic flowering has not yet been extensively studied. Flowering experiments were conducted under the presence or absence of non-damaging UV-B levels. The intensity of the irradiation was selected to simulate natural UV-B conditions, since it was measured as the intensity a plant would receive on a moderately sunny day in Glasgow. The chosen amount of UV-B irradiation did not induce any stress responses which could potentially cause early flowering, as depicted from the low transcript levels of stress gene *COLD-REGULATED 15* (*COR15*) (Lin and Thomashow, 1992; Supplementary Figure 2). From these results it is observed that *Col-0* and *Ler* ecotypes have different stress expression gene responses under UV-B, probably due to their different environmental origins, a common cause for *Arabidopsis* species to develop phenotypic and genetic variations (Alonso-blanco and Koornneef, 2000; Supplementary Figure 2). Our results suggest that out of the three *Arabidopsis* accessions that were tested two of them (*Ler* and *Col-0*) demonstrated an early flowering phenotype when irradiated with low UV-B, more specifically in *Col-0* ecotype a stronger early flowering phenotype was observed compared to a milder one in *Ler* (Figures 1A,B,D,E). On the other hand the third ecotype tested (*Cvi*), depicted a delay in flowering time in response to UV-B (Figures 1C,F). Furthermore, transcript expression analysis of the key flowering regulating genes *FT* and *CO* suggested that a concomitant UV-B dependent upregulation of *FT* and *CO* is observed in *Ler* and *Col-0* ecotypes (Figures 2A–D), while a UV-B dependent downregulation of *FT* occurs in *Cv*i (Supplementary Figure 3). The aforementioned data indicate that different environments of origin are crucial for adaptation of different *Arabidopsis* responses, considering that all three ecotypes originate from very different environmental conditions. *Cvi* is an islandic population (Alonso-blanco and Koornneef, 2000) and Cape Verde islands are known to exhibit a particular geography and a photoperiod of 12 h of light and 12 h of darkness all year long. This contrasts *Col-0* and *Ler* which were originally identified in the United States and Europe, respectively (Koornneef et al., 2010) and are subjected to either a SD (8 h light/16 h darkness) or LD (16 h light/8 h darkness) photoperiod depending on the time of year. Moreover, *Cvi* is generally found in higher altitudes compared to *Col-0* and shorter latitudes compared to *Col-0* and *Ler*, which exhibit very similar altitudes to each other (data acquired from The *Arabidopsis* Information Resource). A possible explanation could be that *Col-0* and *Ler* interpret UV-B irradiation signals as a probable harmful factor even when the intensity of the irradiation is not harmful but could potentially increase, especially if these ecotypes experience a variation of photoperiodic length during the course of a year. In this case early flowering might be triggered as a way for the plant to ensure successful reproduction. On the other hand, ecotypes like *Cvi*, which face little or no changes in photoperiodic length and probably experience less variation in the sunlight they perceive, might have adapted differently in interpreting UV-B irradiation signals. In any case it has been found that it is very common for *Arabidopsis thaliana* species to develop variations both phenotypically and genetically, due to their wide distribution (Alonso-blanco and Koornneef, 2000). Thus, it would be interesting to further examine the genetic variations exhibited in the *UVR8* locus in the *Cvi* ecotype.

Two previous studies reported that under UV-B irradiation both *Ler* and *Col-0* exhibit a late flowering phenotype compared to the corresponding plants grown solely under WL (Hayes et al., 2014; Dotto et al., 2018). The experimental conditions used in the previous studies were significantly different from the ones employed by this research work, as we were interested in testing photoperiodic flowering initiation under a continuous and non-harmful UV-B regime. Previous flowering time analysis conducted in *Col-0* was in response to an irradiation intensity almost 20 times greater than the one utilized in this study, with a duration of 1 h during a SD or LD photoperiod (Dotto et al., 2018). The conditions where *Ler* exhibited a late flowering phenotype under UV-B irradiation, were also different than the growth conditions in our own experiments, since the intensity of the irradiation and the commencement of the UV-B treatment differed; plants were subjected to 9 days of continuous UV-B irradiation after exposure to WL for 10 days (Hayes et al., 2014). The UV-B intensity that was used in the aforementioned study could lead to non-specific stress responses if used long-term. In a more recent study a lower UV-B regime led to a delay in flowering time induction in *Col-0* under a SD photoperiod (Arongaus et al., 2018). Thus, we can conclude that the variability and intensity of the UV-B regime used in flowering time experiments is crucial for inducing flowering initiation, since different UV-B irradiation intensities initiate different UV-B- mediated responses, which subsequently lead to diverse outcomes regarding the initiation of flowering.

Gene expression analysis on the potential mechanism of early flowering initiation further solidifies our results, as both *Col-0* and *Ler* showed significant upregulation of *FT* and *CO* transcript levels under WL supplement with UV-B (Figures 2A–D), suggesting that components of the photoperiodic pathway (Fornara et al., 2010) are involved in the early flowering response.

### Ultraviolet Resistance 8 Acts as a Negative Regulator of Flowering

To better understand if the UV-B receptor UVR8 affects flowering initiation, we investigated flowering time of plants that either lack or over-express UVR8. Our results suggest that UVR8 acts as a negative regulator of flowering under UV-B irradiation. This conclusion was based on the early flowering phenotype that *uvr8* mutant plants demonstrate when grown under WL that was supplemented with UV-B compared to wild type *Ler* (Figure 3A). On the other hand, *uvr8-1* plants flower significantly earlier than wild type *Ler* under WL, while *uvr8-2* mutant plants flower around the same time as *Ler*, suggesting that the type of mutation of UVR8 (*uvr8-1* is a null mutant while in *uvr8-2* the produced protein is impaired) plays a role in their observed flowering phenotype (Figure 3A). These observations are in agreement with our transcript analysis findings, since transcript abundance of both flowering inducers *FT* and *CO* was significantly upregulated in *uvr8* mutants, whilst *FT* levels were significantly downregulated in UVR8 overexpressors (Figures 3B,C), suggesting that CO and FT are involved in UV-B dependent regulation of flowering. Under WL conditions wild type *Ler* and *uvr8-1* mutant plants depict similar transcript levels of *FT* (Figure 3B), with *uvr8-1* flowering earlier (Figure 3A), suggesting that other flowering regulators such as CO are important since *CO* is increased in *uvr8-1* plants compared to wild type *Ler* under WL (Figure 3C). An upregulation of *FT* transcript levels in *uvr8* grown under WL + UV-B compared to WL, has also been observed in a recent study investigating the effects of UV-B in flowering via changes in the activity of the PRC2 complex and miR156 levels (Dotto et al., 2018). The aforementioned research work focused mainly on the potential mechanism that leads to *FLC* upregulation through the control of the age flowering pathway that ultimately delays flowering in their experimental conditions under UV-B irradiation (Dotto et al., 2018).

The significance of our findings is that we provide evidence of an additional flowering pathway in *Arabidopsis*, the photoperiodic, that is involved in the regulation of flowering time under UV-B irradiation, through UVR8-specific mediated responses.

### Ultraviolet-B Accelerates Flowering in Key Flowering and Signaling Mutants

The effect of UV-B on the flowering time of different *Arabidopsis* mutants of key protein components of photoperiodic flowering and/or light signaling, was also analyzed through flowering experiments. UV-B irradiation was found to induce early flowering in *co*, *zlf*, *pif4pif5*, *cop1-4*, and *rup1rup2* leading to the conclusion that these factors may not be essential for UV-B specific acceleration of flowering.

A UV-B induced acceleration of flowering was observed in the late flowering mutants *co* (Wu et al., 2014) and *zlf* (Baudry et al., 2010; Figure 4A). The aforementioned observation for the *co* mutant line would suggest that even if the photoperiodic pathway is most likely involved in UV-B specific acceleration of flowering (Figures 2A–D), it may require additional factors other than CO for the subsequent induction of *FT*. This could imply a putative mechanism where UVR8 bypasses CO in the photoperiodic flowering pathway by interacting directly with the *FT* promoter or by interacting with another transcription factor that induces *FT* expression. This hypothesis is further supported by evidence showing that the *FT* expression is still significantly induced under WL supplemented with UV-B in *co* mutant plants (Supplementary Figure 4).

Another component that has been shown to be involved in flowering induction especially in a temperature-dependent manner is PIF4 (Tavridou et al., 2020). In particular, PIF4 and PIF5 are degraded under UV-B in an UVR8-specific manner (Tavridou et al., 2020). Our results suggest that *pif4pif5* (Lucas et al., 2008) mutants demonstrate the same early flowering phenotype under UV-B conditions as WT (Figure 4B), thus indicating that PIF4 is not essential for UV-B-induced early flowering.

Early flowering time under WL supplemented with UV-B of mutants for UV-B signaling components *cop1-4* (McNellis et al., 1994) and *rup1rup2* (Gruber et al., 2010; Vanhaelewyn et al., 2016) was also induced compared to WL (Figure 4C). RUP2 has been associated with flowering regulation under UV-B conditions (Wang et al., 2011; Arongaus et al., 2018). Previous studies showed that RUP1 and RUP2 play a role in regulating floral transition under WL (Wang et al., 2011). While RUP1 does not regulate flowering, RUP2 was found to repress flowering, under WL conditions (Wang et al., 2011). Intriguingly, over-expression of RUP1 or RUP2 accelerated flowering in plants grown under LD photoperiodic conditions subjected solely to WL irradiation (Wang et al., 2011). *Rup2* mutants demonstrated an early flowering phenotype as well as RUP2 over-expressing plants, indicating a more complex regulatory mechanism affecting photoperiodic flowering, whilst both factors were found to be controlled by the circadian clock (Wang et al., 2011). A more recent study indicated that *rup2* mutants flower at the same time as WT under WL LD conditions (Arongaus et al., 2018), a phenotype similar to our findings where under WL *rup1rup2* flowering time is not statistically different to the one of wild type *Col-0* (Figure 4C). Under SD WL supplemented with UV-B, *rup2* and *rup1rup2* flower earlier than wild type plants in a UVR8 dependent manner (Arongaus et al., 2018). Interestingly, this observation correlates with our own findings from flowering experiments conducted under a LD photoperiod for *rup1rup2* (Figure 4C). Moreover, it was demonstrated that RUP2 and CO can interact directly and that the early phenotype of *rup2* is dependent on both *FT* and *CO* (Arongaus et al., 2018), suggesting that factors of the photoperiodic flowering pathway are indeed essential for mediating the early UV-B dependent flowering initiation response under both LD and SD photoperiods.

### Ultraviolet-B Delays the Early Flowering Phenotype of *elf3-1* and OXPIF4

In contrast to the other genotypes examined, *elf3-1* (Zagotta et al., 1992) and OXPIF4 (Kumar et al., 2012), demonstrated a slight delay in flowering initiation under WL + UV-B compared to WL (Figures 4D,E). This data would suggest that ELF3 could potentially act as a key regulator of the UV-B mediated flowering initiation response downstream of UVR8. Our results altogether indicate that UV-B has clearly a different effect on flowering initiation of early flowering mutants. Thus, it would be particularly interesting to further examine the molecular mechanism underlying these responses and examine the effect of UV-B on additional early flowering [possibly *SUCpro*::CO-HA also known as the over-expressing CO line (Mizuno et al., 2014)]. Flowering time regulation is a very complex process regulated by many different protein factors and pathways (Fornara et al., 2010), therefore it is possible that in mutant or transgenic plants exhibiting an early flowering phenotype under WL, UV-B signal affects differently photoperiodic flowering responses.

**FIGURE 5:**
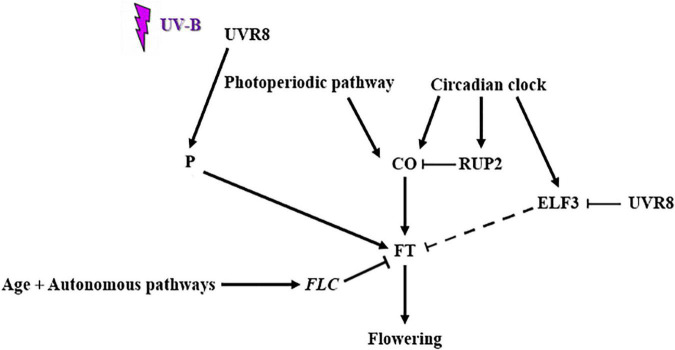
Schematic representation of UV-B mediated control of flowering time (factors and flowering pathways involved). Upon UV-B irradiation multiple flowering pathways control flowering time. The age and autonomous pathways lead to an upregulation of *FLC* [as demonstrated by Dotto et al. (2018)], which acts as an *FT* repressor. CO and FT are flowering promoting factors of the photoperiodic pathway. CO is repressed by RUP2, a negative regulator of UVR8 signaling [as demonstrated by Arongaus et al. (2018)] and a circadian clock component. ELF3, another circadian clock component also represses *FT*, indirectly by targeting CO for degradation. UVR8 represses *ELF3* and possibly interacts with another protein factor (P) in order to promote *FT* gene expression.

Based on all of the above conclusions a preliminary and simplified model of UVR8 action can be formulated (Figure 5). More specifically upon UV-B irradiation multiple flowering pathways control flowering time. The age and autonomous pathways lead to an upregulation of *FLC* (Dotto et al., 2018), which acts as an *FT* repressor. CO and FT are flowering promoting factors of the photoperiodic pathway. CO is repressed by RUP2, a negative regulator of UVR8 signaling (Arongaus et al., 2018). ELF3, a circadian clock component also represses *FT*, indirectly by targeting CO for degradation. UVR8 represses *ELF3* and possibly interacts with a protein factor that has not yet been identified in order to promote *FT* gene expression. However, further investigation need to be performed in order to identify the mechanism and factors that are involved in the UV-B specific control of flowering time in *Arabidopsis thaliana*, through the action of UVR8.

Our experiments have provided further insights on the way UV-B irradiation affects flowering initiation in *Arabidopsis thaliana*. However, future experiments need to be performed in order to fully elucidate the molecular mechanism, as well as the physiological significance of these flowering time alternations. Uncovering the mechanism underlying UV-B-mediated flowering initiation, including all the factors and pathways that integrate the UV-B-specific photoperiodic flowering, as well as determining the role of UVR8 in this process, will further advance our abilities on designing more efficient agricultural practices which will contribute to the goal of improving global food security. Developing strategies to improve crop resilience and increase yield according to environmental stimuli such as UV-B, can provide the tools to maximize global crop production. Deciphering these mechanisms primarily in *Arabidopsis thaliana* consists an integral part of this process since it is the most universally used model plant organism, due to its many advantages and the ability to identify homologs in many commercial crops.

## Data Availability Statement

The original contributions presented in the study are included in the article/Supplementary Material, further inquiries can be directed to the corresponding author/s.

## Author Contributions

AZ and EK designed and performed the research, and analyzed the data. AZ, EP, and EK wrote the manuscript. AZ performed genotyping, western blot analysis, and all qRT-PCR experiments and analysis. EP performed RNA extraction and assisted with flowering experiments. LO’D assisted with the performance of flowering experiments. All authors contributed to the article and approved the submitted version.

## Conflict of Interest

The authors declare that the research was conducted in the absence of any commercial or financial relationships that could be construed as a potential conflict of interest.

## Publisher’s Note

All claims expressed in this article are solely those of the authors and do not necessarily represent those of their affiliated organizations, or those of the publisher, the editors and the reviewers. Any product that may be evaluated in this article, or claim that may be made by its manufacturer, is not guaranteed or endorsed by the publisher.
