# Supplementary figures and images for: Low Fluence Ultraviolet-B Promotes Ultraviolet Resistance 8-Modulated Flowering in Arabidopsis
Source: Front Plant Sci. 2022 Mar 31;13:840720. doi: 10.3389/fpls.2022.840720 (PMC9009151; doi:10.3389/fpls.2022.840720)

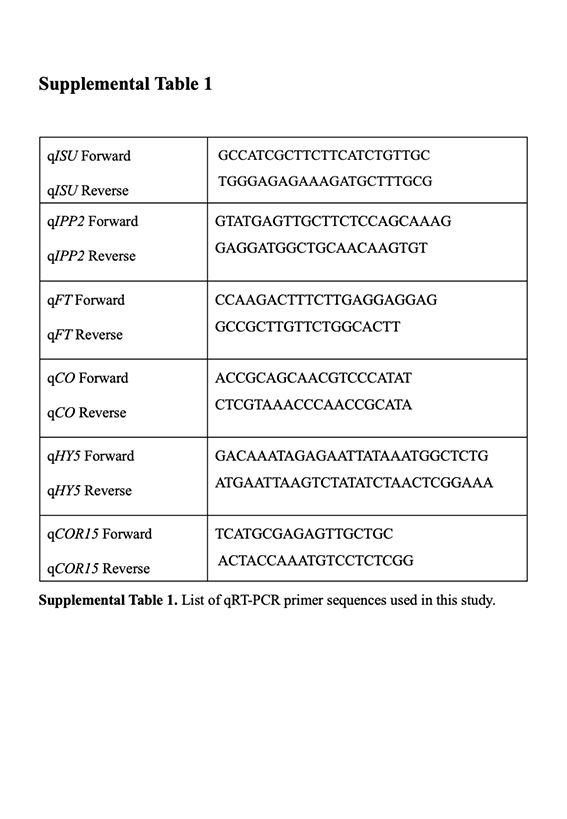

Supplement: Supplementary file 1 [file Image_1.TIFF]

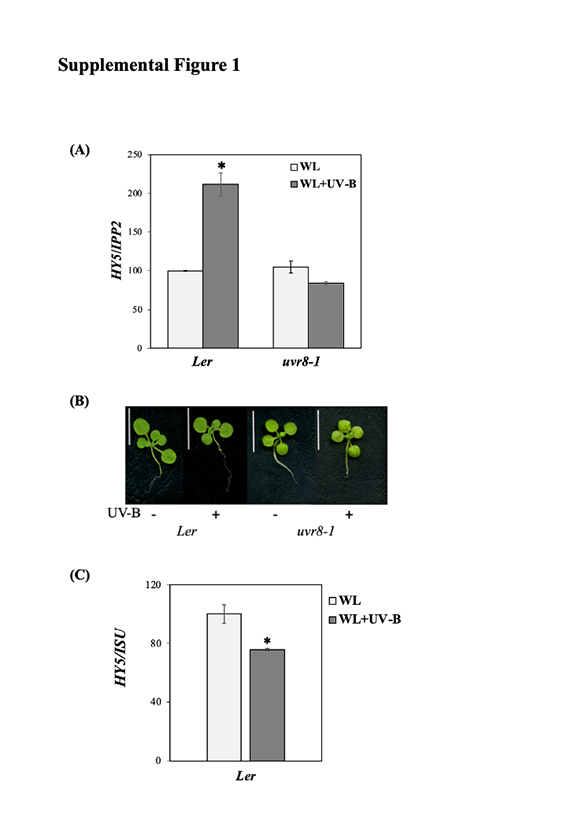

Supplement: Supplementary file 2 [file Image_2.TIFF]

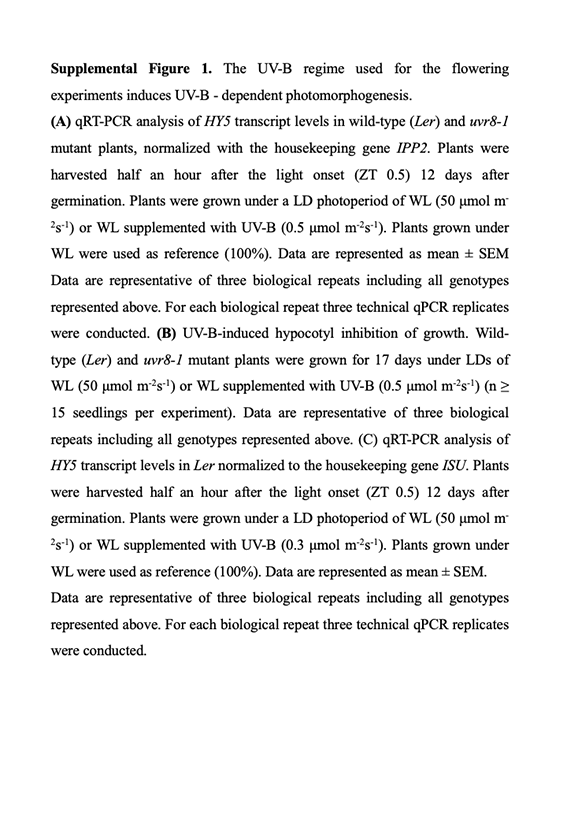

Supplement: Supplementary file 3 [file Image_3.TIFF]

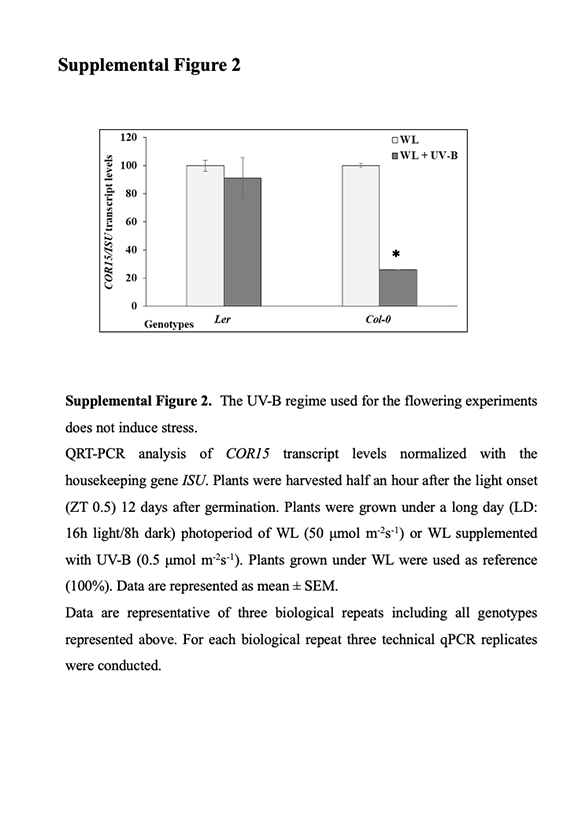

Supplement: Supplementary file 4 [file Image_4.TIFF]

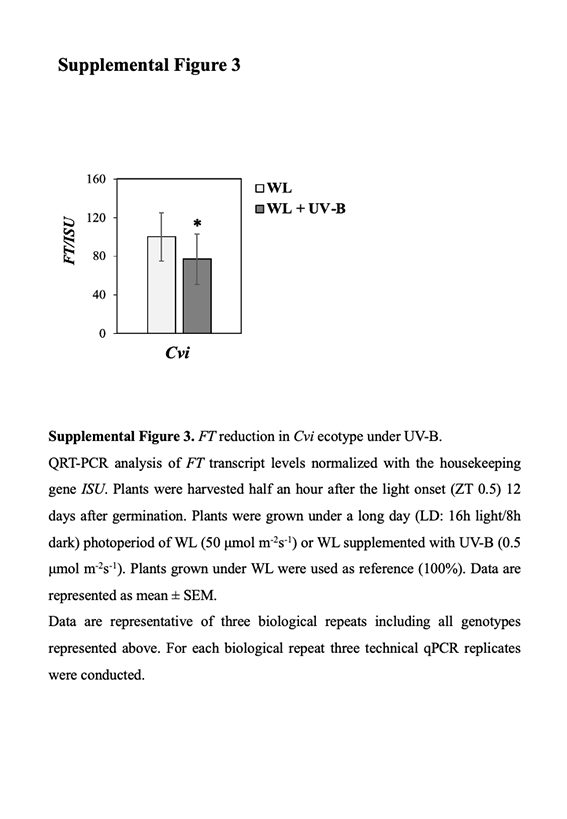

Supplement: Supplementary file 5 [file Image_5.TIFF]

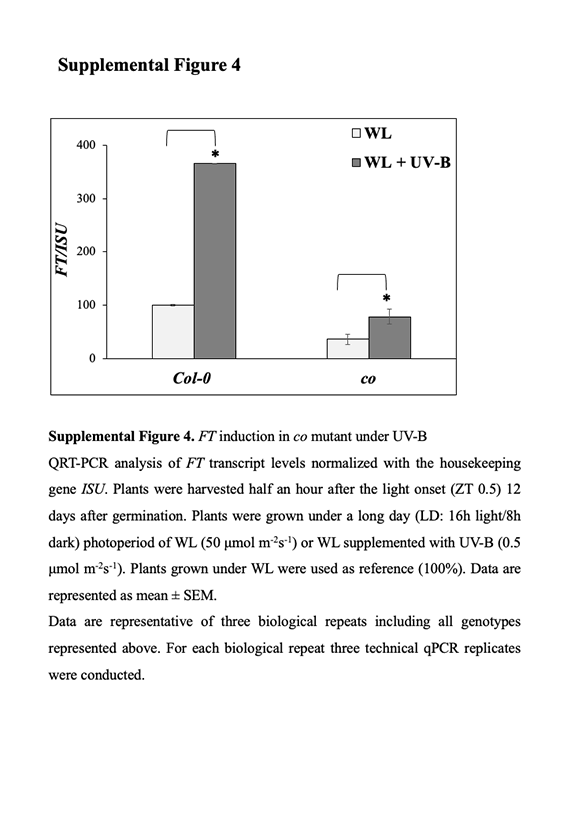

Supplement: Supplementary file 6 [file Image_6.TIFF]
